# Education as an enabler, not a requirement: ensuring access to self-care options for all

**DOI:** 10.1080/26410397.2022.2040776

**Published:** 2022-03-03

**Authors:** Caitlin Corneliess, Katelin Gray, Jennifer Kidwell Drake, Allen Namagembe, Aurora Stout, Jane Cover

**Affiliations:** a Director, DMPA-SC Access Collaborative, PATH, Washington, DC, USA. *Correspondence*: ccorneliess@path.org; b (Former) Program Officer, PATH, Washington, DC, USA; c (Former) Team Lead, Sexual and Reproductive Health, PATH, Seattle, WA, USA; d Deputy Project Director, Uganda Self-Injection Scale-Up, PATH, Kampala, Uganda; e Program Officer, PATH, Seattle, WA, USA; f Research and Evaluation Manager, PATH, Seattle, WA, USA

**Keywords:** self-care, self-injection, sexual and reproductive health and rights, family planning, contraception, education, health literacy, decision-making, autonomy

## Introduction

Around the world, new innovations and access to information are making it easier for people to have greater autonomy over their sexual and reproductive health and rights (SRHR). Many governments and health providers are increasingly taking action to support long-standing self-care practices, ensuring connections to the health system where needed. For SRHR self-care options to be equitable and accessible to everyone, their design and implementation must account for varying levels of formal education and literacy among users.^1^

Self-care can enable individuals to preserve their health and treat illness, either with or without the support of a health care provider. Expanding self-care options can increase individuals’ autonomy, improve health outcomes, and close the equity gap in health services. In 2021, the World Health Organization (WHO) released a second guideline on self-care interventions for health and well-being, which is rooted in a review of global evidence and SRH-related human rights, including “the right to be informed of and have access to the safe, effective, affordable and acceptable methods of fertility regulation of their choice”.^1^ These rights extend to all populations that may experience stigma, discrimination, or other barriers when accessing health services, including individuals who have not completed primary or secondary education. In a recent commentary on discrimination in health care, for example, Babyar writes, “Providers may tailor conversations and wording based on an individual’s work or education achievements, displaying either negative or positive discriminatory practice”.^2^

The WHO guideline’s conceptual framework recognises that both information and education are enabling factors for practising self-care. Users must have access to accurate, high-quality information tailored to different education and literacy levels.^1^ At the same time, there is a risk that some policy-makers, implementers, and health providers may consider higher levels of education to be a requirement for practising self-care, rather than an enabler – a risk that has played out in discussions about self-care interventions including contraceptive self-injection (see below) as well as HIV self-testing.^3^ This risk would disproportionately affect women and girls because of long-standing gender disparities in education,^4^ as well as pervasive gender norms that can undermine trust in women’s and girls’ autonomy and decision-making. To address real and potential disparities, we must continue to advance inclusive and equitable quality education for all, in alignment with the Sustainable Development Goals (SDGs). The SRHR community must also take deliberate action to design and implement self-care interventions that account for varying levels of education, literacy, and numeracy.

In this commentary, we advocate for self-care options that are equitable and accessible to everyone, drawing from experiences in introducing self-injectable contraception.

## The role of information, education, and health literacy in enabling self-care

While the evidence base exploring the interaction between education level and the practice of self-care for SRHR is still emerging, it is well established that formal education contributes to improved SRHR outcomes. In countries with large disparities in educational attainment, modern contraceptive use is between 30% and 70% higher among women with primary or secondary education than among women without formal education.^4^ Furthermore, education is closely linked to health literacy, autonomy, and agency, all enabling factors for practising self-care.^1,4^ Education also mediates multiple other vulnerabilities: impacting wage earning, helping avert child marriage, and contributing to conflict prevention.^5^ As self-care expands as a delivery strategy, historical SRHR access barriers already faced by women with limited formal education may be compounded by the fact that stakeholders, including providers, have expressed concern about the ability of individuals with limited education to use self-care options safely and effectively. These concerns may be magnified as the complexity of self-care interventions increases along the continuum of available options.

## Spotlight on self-injection: stakeholder perceptions regarding education and self-care

Self-injection of subcutaneous depot medroxyprogesterone acetate (DMPA-SC) is a key self-care intervention recommended in the WHO self-care guidelines and has been the subject of considerable evidence generation, including helpful insights on the role of education in self-care practices. Because DMPA-SC is prefilled and easy to inject, anyone can administer it following a brief training, including family planning clients themselves. Evidence also shows that self-injection is acceptable among women in a wide range of countries and can increase continuation rates and reach first-time users of modern contraception.^1^ Self-injection has been approved in more than 32 countries and is being rolled out through public facilities, private outlets like pharmacies and drug shops, and at the community level.

As countries prepared to introduce self-injection, qualitative interviews conducted by PATH* and partners with family planning decision-makers in Burkina Faso, Niger, Senegal, and Uganda in 2014–2016 revealed that participants from each country expressed concerns or hesitation about clients’ level of education.

In Burkina Faso, some stakeholders said that educated women might be able to self-inject competently, yet they felt that low levels of education and literacy – especially among rural women – pose a significant barrier to self-injection: “My problem with self-injection – I come back to this – is the level of education of women” (Ministry of Health representative, Burkina Faso). Many participants in Niger felt that despite the practice’s evident advantages, the country was not yet ready to offer self-injection due to low overall levels of education and literacy: “Self-injection is for people in cities, it is for rich people, or people more or less educated” (Intergovernmental agency representative, Niger). In Senegal, stakeholders emphasised the need for more data on self-injection among women by education level:

*“I would like to know the proportion of women who choose this method – their age, their education level. Because certainly, the one who is uneducated, when you tell her how to do the injection, it’s not for sure that she will understand right away how easy it is.”* (Ministry of Health representative, Senegal)

And in Uganda, respondents tended to address the topic indirectly, noting that early adopters might be more-educated women and university students.^6^

These interviews reveal an uncomfortable paradox faced by the SRHR community in considering the connection between self-care and education: while self-care options have the potential to benefit women with less education or literacy who often face greater barriers in accessing SRH information and services generally, education is perceived as a prerequisite for self-care by some critical decision-makers, including some health care providers. A number of studies and programming experiences discussed in this article offer insight into how education may inform a woman’s potential self-injection journey: how she learns about self-injection, whether health workers offer her a full range of options, whether she has the confidence to self-inject successfully, and whether she continues the method in alignment with her family planning intentions. This evidence may help us chart a path toward equitable access to self-care for women with less formal education.

We have chosen to highlight the example of self-injection in this article because of the extensive recent evidence on its use among diverse populations relative to other self-care interventions, which include both products and behaviours. Lessons from self-injection may not be universally generalisable across self-care options, including due to issues such as complexity of instructions or novelty, as well as other attributes (e.g. practices like injections may come with more fear than oral pills). Where possible, we have tried to highlight limitations and evidence gaps to be addressed in terms of generalisability.

## Spotlight on self-injection: summary of results to date among women with less education

Governments and implementing partners in Malawi, Senegal, and Uganda were among the first to rigorously study and offer contraceptive self-injection as a self-care option for women. Evidence from a randomised controlled trial in Malawi has shown that women with limited to no formal education are successful with self-injection. Most women enrolled in the study had less than primary school education (whether in the self-injection or provider administration group), and there was no difference in continuation rates by educational attainment.^7^ In addition, health worker perceptions collected during a qualitative ancillary study indicate support for self-injection by women with less education: as one said, “ … where I am here, a lot of people are primary school dropouts. They can't even write, but they are the ones you will find to be self-injectors and are managing to inject themselves”.^8^

Results from Senegal and Uganda vary from the Malawi experience in some ways. Evidence from Senegal and Uganda has shown that women with less education needed additional support to both initiate and continue to self-inject. In both countries, in year-long prospective cohort studies where women visiting family planning clinics for injectable contraception were given the choice to self-inject or receive an injection from a provider, women who chose self-injection were more likely to have a secondary education or higher.^†^ Additionally, having a primary or secondary education decreased the probability a self-injector would discontinue the method during the one-year period.^9,10^ In Senegal in particular, this may relate to challenges in remembering the reinjection window: forgetting to reinject or reinjecting late represented nearly half of the discontinuation cases among women in the study.

Results from a follow-on self-injection programme evaluation in Uganda may yield insights into programme strategies for women who struggle to learn to self-inject correctly. These programme strategies were co-designed with input from clients, providers, district health leaders, and national decision-makers. The evaluation of that (routine service delivery, not research) programme found that clients with more education were better able to demonstrate injection competency, but factors reflective of their training, including training in the use of the job aid, were more important (unpublished observation).

## Recommendations for programmes to ensure access to self-care for all, regardless of education

Access to self-care products and practices should be reinforced, not restricted, for people with less education. Health workers and family planning decision-makers at every level can ensure programmes provide all people with the information, training, and support necessary to practice self-care interventions successfully – accounting for the complexity of different self-care interventions, which varies considerably. The recommendations below are likely to be especially important for relatively more complex self-care interventions, but the varying results from self-injection research to date also help highlight that policy-makers and providers should not make blanket assumptions about what people can and cannot learn to do, based on a variety of background characteristics including education. Such assumptions could well result in inequitable access to self-care options. At the same time, the wide range of individuals who have learned to self-inject across geographies offers hope for other new self-care interventions. In research and evidence-gathering, self-care programmes should continue to collect, analyse, and share data on self-care experiences among women with limited education and/or literacy and numeracy in order to further inform strategies for ensuring equitable access to SRH self-care for all.

New and evolving self-care frameworks must explicitly address populations with less formal education and the requirements for reaching them. The Self Care Trailblazer Group’s Quality-of-Care Framework for Self-Care^11^ indicates that clients should be able to access “care or information that does not vary in quality because of their personal characteristics such as age, marital status, gender, disability, ethnicity, geographic location, and socioeconomic status” (Standard 4.3).^11^ We would like to expand on this standard and advocate that it is worth stating explicitly that information should not vary in quality across additional characteristics, including educational background, literacy, and numeracy. The client communication tools channel of the framework emphasises information in local languages (Standard 3.3) and could go further to include pictorial, video, or audio options. Special attention should be paid to the literacy levels of the audience when designing programme tools like job aids for clients, by simplifying technical and text-heavy content. Such modifications to the standards to recognise education level could ensure high-quality rights-based self-care for those with lower levels of education or literacy and prevent further deepening inequities.

As another example, the Digital Self-Care Framework from the Self-Care Trailblazer Group instructs family planning programme managers to “consider the existing foundation of health literacy and comprehensive health education in the population to create a supportive environment for individuals to engage in self-care through digital channels”.^12^ Digital self-care approaches in settings with variable education, literacy, and numeracy levels – and access to digital tools – should always be complemented and reinforced by multiple/different training and outreach approaches to ensure digital self-care that is not easily accessible to everyone does not further exacerbate disparity. For example, group information sessions or training on self-care practices facilitated by a health worker could be paired with digital health interventions. In early self-injection rollout in Uganda, women reported that provider-led group trainings on self-injection offered more comprehensive training content because some clients asked questions others did not think about or feel comfortable asking. Group training can also facilitate the development of “buddy systems” for self-care peer support in the future, as well as potential troubleshooting on digital technologies or sharing of digital tools and information (unpublished observation). The importance of multiple approaches to meeting clients’ intersecting needs is only reinforced by the fact that group training may not be appealing to some, including adolescent and youth clients or others who prefer to keep contraceptive use private.

Similarly, health systems and providers themselves must address structural and personal biases and reinforce rights-based informed choice, meeting all clients where they are. Health providers may be reluctant to embrace self-care interventions for a variety of reasons: for example, injecting may be considered the provider’s expertise, or a self-care intervention may be perceived as too hard. Providers must spend enough time with clients to ensure they master self-care practices and have the confidence to act independently. However, provider time constraints due to workload can magnify provider reluctance to offer any self-care option that requires additional time or client training, and this may be especially true for less educated women who may have real or perceived needs for more counselling and training time. Involving group training or use of visual aids and videos may be tools that help busy providers find time for this. These approaches are being evaluated currently, and results are forthcoming, yet even more work needs to be done with health care providers to co-create solutions on embracing self-care. At the same time, encouraging and equipping women who are competent and feel confident to initiate self-injection alone may also prioritise health workers’ resources for women who need more practice and support from the health system. Experience in Uganda showed that women using training videos can learn to self-inject entirely independently, especially those with higher levels of education (unpublished observation).

Missing the reinjection window was a common reason for discontinuation reported by study participants in Senegal and Malawi. The challenges with tracking reinjection dates experienced in Senegal suggest a need to assess provider counselling practices around self-care interventions that require numeracy, which could easily be taken for granted in self-care programming. Health providers, especially community health workers, can provide tailored reminders or follow-up support on such skills with clients in person or through cell phones, audio messages, or SMS (taking literacy into account). Again, overlapping vulnerabilities need to be considered, and clients must explicitly confirm that they intend to continue voluntarily and give advance permission to be contacted, considering that covert contraception use is practised by many women.

Finally, our primary focus is on how education level mediates access to self-care, but we recognise that gender and gender identity can also contribute to variable health and educational outcomes. Self-care options, for example, can provide greater access to contraception for those who may feel marginalised by or excluded from the health system based on their gender identity, including queer, trans, and nonbinary individuals. Due to the gender-binary nature of available data on education and contraceptive use, we refer to “women and girls” within this article. We recognise, however, the limitations of binary language, and moving forward, we call on the public health sector to expand language and data collection efforts to be inclusive of all gender identities.

The global community continues to work toward inclusive and equitable quality education, in alignment with SDG 4, but access to self-care for all cannot wait until we realise this goal. Strategies which empower, train, and support women who have limited formal education to practice self-care can ensure that these options are within reach of all women, regardless of their education level.

## Data Availability

Data are available on request from the authors.

## Footnotes

* PATH is a global nonprofit dedicated to achieving health equity. Two authors (AS and JC) of this commentary were researchers on these qualitative studies. The studies were led by PATH in all four countries, and in Niger and Burkina Faso conducted in collaboration with United Nations Population Fund.

† Because the Malawi study was a randomised trial design, we do not know if women with more education would have self-selected into self-injection if they had the choice.
